# Targeting the undruggable oncogenic KRAS: the dawn of hope

**DOI:** 10.1172/jci.insight.153688

**Published:** 2022-01-11

**Authors:** Hande Asimgil, Utku Ertetik, Nedim Can Çevik, Menar Ekizce, Alper Doğruöz, Muazzez Gökalp, Elif Arık-Sever, Rouzanna Istvanffy, Helmut Friess, Güralp Onur Ceyhan, Ihsan Ekin Demir

**Affiliations:** 1 Department of Surgery, Klinikum rechts der Isar, Technical University of Munich, School of Medicine, Munich, Germany.; 2 Department of General Surgery, Hepatopancreatobiliary-Unit, School of Medicine, Kerem Aydınlar Campus at Acıbadem University, Istanbul, Turkey.; 3 German Cancer Consortium (DKTK), Partner Site Munich, Munich, Germany.; 4 SFB/Collaborative Research Centre 1321 Modelling and Targeting Pancreatic Cancer, Munich, Germany.; 5 Else Kröner Clinician Scientist Professor for Translational Pancreatic Surgery, Munich, Germany.

## Abstract

KRAS mutations are the drivers of various cancers, including non–small cell lung cancer, colon cancer, and pancreatic cancer. Over the last 30 years, immense efforts have been made to inhibit KRAS mutants and oncogenic KRAS signaling using inhibitors. Recently, specific targeting of KRAS mutants with small molecules revived the hopes for successful therapies for lung, pancreatic, and colorectal cancer patients. Moreover, advances in gene editing, protein engineering, and drug delivery formulations have revolutionized cancer therapy regimens. New therapies aim to improve immune surveillance and enhance antitumor immunity by precisely targeting cancer cells harboring oncogenic KRAS. Here, we review recent KRAS-targeting strategies, their therapeutic potential, and remaining challenges to overcome. We also highlight the potential synergistic effects of various combinatorial therapies in preclinical and clinical trials.

## Introduction

KRAS is a frequently mutated proto-oncogene that drives epithelial-mesenchymal transition, which leads to tumorigenesis mainly in the lung, colon, and pancreas (1, 2). KRAS belongs to the human *RAS* gene family that encodes three small GTPases (NRAS, HRAS, and KRAS) that cycle between GTP-bound active and GDP-bound inactive states. KRAS is located on the inner leaflet of the plasma membrane, and active KRAS transduces extracellular signals from receptor tyrosine kinases (RTKs) to downstream signaling pathways, thus controlling cell proliferation, differentiation, transformation, and apoptosis (3). The GTP/GDP molecular switch takes place upon translocation of GEFs and GAPs toward the proximity of KRAS (4). The mutations in the GTP-binding site confer resistance to GTP hydrolysis by GAPs, resulting in constitutively active KRAS (5). Hyperactive KRAS induces oncogenic transformation by upregulating downstream signaling pathways, including PI3K/AKT/mTOR, RAF/MEK/ERK, MAPK/ERK, and RALGEF/RAL (6).

Although RAS proteins exhibit some structural homology and share similar functional and biochemical properties, the oncogenic potential of each RAS isoform varies by the tissue, codon, substitution type, and mutation frequency. More than 80% of mutations in KRAS occur at codon 12, found prevalently as G12D substitution in 70% of pancreatic ductal adenocarcinoma (PDAC) (7) and in almost 50% of colorectal carcinoma (CRC) cases (8). On the other hand, G12C is harbored more frequently in non–small cell lung carcinoma (NSCLC) and is present in approximatively 40% of metastatic lung adenocarcinoma cases (9). From a clinical perspective, KRAS mutants are attractive potential therapeutic targets (10). Thus, numerous efforts have been made over the last 30 years to inhibit mutant KRAS with small molecules. However, attempts to develop GTP analog inhibitors have been challenged by the structural properties of the GTP-binding pocket, high homology between RAS proteins, high affinity between GTP and KRAS, and high concentration of GTP in cells in vivo (11, 12). Alternatively, intensive investigations have been made toward targeting downstream KRAS effectors, including the RAS-binding domain of RAF, the MAPK pathway effector kinases MEK and ERK, and mTOR of the PI3K/AKT pathway (see refs. 12–15 for recent reviews on efforts targeting these pathways).

In the last decade, novel approaches involving the harnessing of the patient’s immune system have been introduced as alternatives to standard therapies. Checkpoint inhibitors, cancer vaccines, and adoptive cell therapies are being introduced to promote antitumor immunity to recognize and kill cancer cells more efficiently. In addition, next-generation KRAS small-molecule inhibitors are making their way into clinics, either as monotherapy or in combination with other therapies, and these developments provide an alternative highway to fight hard-to-treat KRAS-driven cancers. Here, we outline emerging direct targeting and immunotherapeutic strategies, highlight results of corresponding preclinical and clinical trials in the context of lung, colorectal, and pancreatic cancer, and focus on clinical applications targeting oncogenic KRAS proteins. We also discuss combination strategies that hold great potential for personalized treatment and durable clinical benefits.

## Direct targeting of oncogenic KRAS

### Allele-specific small-molecule inhibitors.

All-in-one strategies to directly inhibit all KRAS alleles with GTP analogs that target the GTP-binding site failed in clinical trials in recent decades (12, 16). Nevertheless, allele-specific inhibitors designed on the basis of structure have become game changers in oncogenic KRAS–driven cancer therapies.

Shokat and colleagues, who focused on the KRAS G12C allele because of its relatively reactive 12-Cys residue and high intrinsic GTPase activity, discovered an additional allosteric pocket (S-IIP) on KRAS under the switch-II loop region, which is only accessible in the GDP-bound conformation (17); thus, targeting this allosteric region with a covalently bound small molecule locks GDP-bound KRAS G12C in the inactive state. This discovery was followed by development of a new series of small molecules; most showed insufficient potency in in vitro and in vivo settings (17–20). Nevertheless, study of ARS-1620, which selectively binds GDP-KRAS G12C, disclosed His95 in the vicinity of S-IIP and showed potential for therapeutic use in preclinical studies (21). Structurally similar to but more potent and selective than ARS-1620, AMG510 (sotorasib) and MRTX849 (adagrasib) were found through screening of His95 groove-binding molecules (22, 23). Sotorasib (ClinicalTrials.gov NCT03600883) and MRTX849 (NCT03785249) are being evaluated in phase I/II clinical trials in patients with advanced KRAS G12C–mutant solid tumors, particularly those with NSCLC. Sotorasib has been evaluated both as monotherapy and in combination with anti–PD-1/PD-L1. In phase I trials, the drug showed promising anticancer activity without causing dose-limiting toxic effects or treatment-related deaths among 129 patients (59 with NSCLC, 42 with CRC, and 28 with other tumors) who had received standard therapies. The primary safety endpoint was achieved, as only 11.6% of patients showed severe treatment-related adverse effects (24). In a subgroup of patients with KRAS G12C–induced advanced NSCLC who were treated daily with oral sotorasib in a phase II trial, 37.1% exhibited objective, 3.2% complete, and 33.9% partial responses, with a medium duration of 11.1 months (25).

MRTX849 exhibited broad-spectrum antitumor activity among NSCLC and CRC patients and was tolerable at high doses in early clinical trials (26). However, some patients given MRTX849 monotherapy acquired drug resistance. The comparative single-cell sequencing of patient tumor samples before and after treatment demonstrated that a subgroup of cancer cells acquire diverse drug resistance mechanisms involving EGFR, SH2-containing protein tyrosine phosphatase-2 (SHP2), and aurora kinase signaling to maintain newly produced KRAS G12C in the active state (23, 27). Evidently, one NSCLC patient who developed acquired resistance to MRTX849 after 4 months of treatment developed ten individual alterations, primarily in genes that reactivate RAS/MAPK signaling, including a KRAS Y96D mutation that is insensitive to G12C inhibitors (28). Furthermore, by applying comprehensive genetic and histological analysis to the MRTX849-resistant tumor samples from 38 patients with NSCLC, CRC, and appendiceal cancer, Awad et al. released a list of alterations, including the oncogenic mutations of KRAS and other RAS proteins, KRAS G12C allele amplifications, loss-of-function mutations in genes regulating cell growth and division, and oncogenic fusions of RTKs to feed back RAS/MAPK pathway (29).

In order to attain durable and broad antitumor response, phase Ib clinical trials have been conducted in several arms to evaluate combinatorial treatments of MRTX849 with such inhibitors, afatinib (RTK inhibitor in NSCLC patients), cetuximab (EGFR inhibitor in metastatic CRC patients), and pembrolizumab (PD-1/PD-L1 inhibitor in NSCLC patients). Studies of MRTX849 and pembrolizumab combination modalities are also in progress to evaluate clinical efficacy, safety, tolerability, and duration of response in NSCLC patients with KRAS G12C (NCT04613596). Similarly, phase I/II clinical trials are ongoing of two novel KRAS G12C–targeting small molecules developed by Roche (NCT04449874) and InventisBio (NCT04585035) as single agents and in combination with EGFR, VEGFA, PD-L1 inhibitors, and standard treatments. Remarkably, inhibition of the nonreceptor protein tyrosine phosphatase SHP2, which works as a hub between RTKs and RAS proteins, in preclinical studies resulted in sensitization of KRAS-mutant cells to RAS/MAPK effector–targeting inhibitors (30, 31). Therefore, phase I/II trials to evaluate the therapeutic potential of SHP2 inhibitors in combination with MRTX849 (NCT04330664) are under way. Based on the knowledge gained from G12C inhibitor studies, novel inhibitors that target the other prevalent KRAS alleles are being explored in preclinical models. Mirati Therapeutics declared that their inhibitor MRTX1133 is designed to inhibit the G12D allele in both active and inactive states, and MRTX1133 has promise for clinical translation due to its long half-life, potency, allele selectivity, and antitumor activity in preclinical trials (32).

### RNAi technology: overcoming the barrier of instability.

RNA interference (RNAi) has been utilized in the last two decades to silence expression of oncogenes and oncogenic signaling effectors and is highly potent in cancer therapies (33). In the earliest studies, Brummelkamp et al. reported that it was possible to specifically and stably silence a targeted gene product with siRNA in vitro using virus- and plasmid-based systems (34). Furthermore, they also showed that suppression of KRAS G12V by retroviral siRNA caused loss of tumorigenicity of pancreatic cancer cells harboring KRAS G12V in a subcutaneous xenograft, proving that siRNA has power as a tumor-specific gene therapy tool (35). Multiple developments in delivery formulations and the chemical structure of the oligonucleotides were made to enhance siRNA efficacy in targeting oncogenic KRAS in lung, colon, and pancreatic cancer cells. The KRAS antisense oligonucleotide AZD4785 from AstraZeneca failed in a phase I clinical study owing to significant concerns about the lack of efficacy, although the oligonucleotide was proven to be safe and well tolerated in preclinical murine and nonhuman primate studies (36–38). The most recent effort to use siRNA as therapeutic tool was made by Strand et al., who used the autochthonous pancreatic cancer mouse model KPC (*LSL-Kras^G12D^*, *p53^lox/lox^*, *p48^Cre/+^*) and showed that precise and effective delivery of siRNA coated with peptide-based nanoparticles to the tumor microenvironment (TME) promotes tumor regression (39).

**Figure 1:**
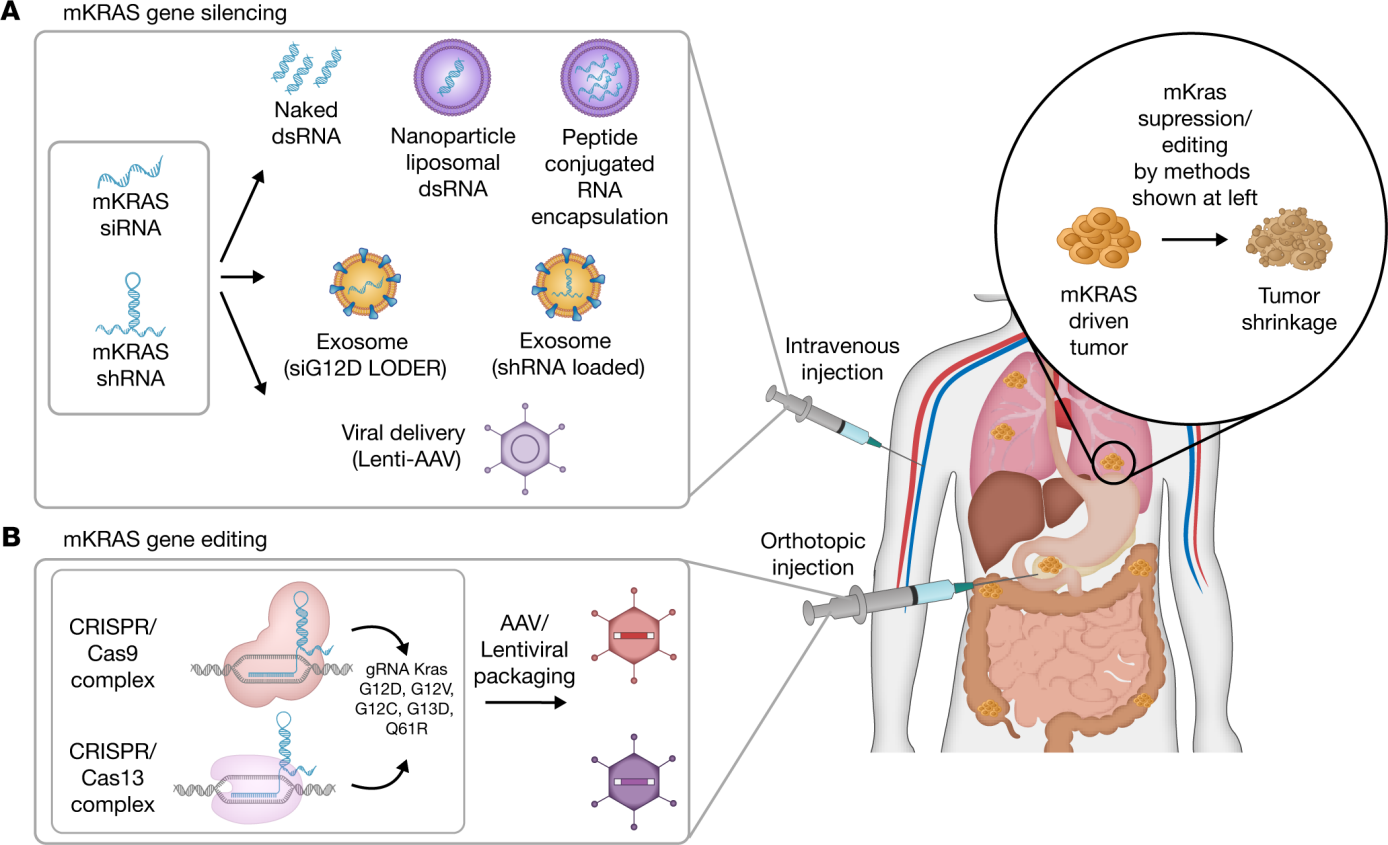
RNAi and CRISPR technology for treatment of oncogenic KRAS–driven cancers. (**A**) Mutant KRAS–specific (mKRAS-specific) siRNAs and shRNAs silence the expression of mKRAS by generating an RNA hybrid complex that induces endogenous *KRAS* mRNA degradation. Inhibitory RNA molecules are encapsulated in a liposome, exosome, or nanoparticle and can be administered to patients via intravenous injection or orthotopic injections for access to oncogenic KRAS–driven tumor sites. Alternatively, adeno-associated viral (AAV) vectors may be used to intravenously deliver mKRAS-targeting therapeutics. Despite major safety concerns, viral vector delivery systems (adenoviral, retroviral, and lentiviral vectors) provide longer-lasting effects on RAS hotspot mutations. (**B**) Viral delivery of CRISPR/Cas9 (DNA) or Cas13 (RNA) systems that target mKRAS-expressing tumor cells is administered to the tumoral site via orthotopic injection. Through administration of KRAS sgRNAs in the CRISPR/Cas13 system, only transient correction of cancer cells at the post-transcriptional level can be attained. By directly targeting mKRAS, both RNAi- and CRISPR-based therapeutics promote tumor reduction.

Silenseed Ltd. provided a remarkable development in the field with their Local Drug EluteR (LODER) system, in which a KRAS G12D–targeting siRNA was embedded in a biodegradable polymeric matrix (siG12D-LODER). In a phase I/II study (NCT01188785), siG12D-LODER was implanted directly into the tumor site of patients with locally advanced pancreatic cancer with a standard endoscopic ultrasound-guided biopsy needle in combination with chemotherapy (gemcitabine or FOLFIRINOX) (Figure 1 and ref. 40). Evaluation of the primary outcome revealed that the therapy improved progression-free survival in the study population in a one-year time frame without causing dose-related toxicity (40). A phase II study (NCT01676259) has been evaluating this biodegradable siG12D-bearing miniature drug administered as a single dose at 12-week cycles in combination with gemcitabine plus nab-paclitaxel in 80 participants (41).

iExosomes are stromal cell–derived, engineered vesicles that contain KRAS G12D siRNA and present CD47 protein on their surface, which increases persistence of the vesicles in the body, as CD47 is known to prevent phagocytosis by macrophages and monocytes (42, 43). In addition, the design takes advantage of enhanced micropinocytosis of iExosomes only by oncogenic KRAS–transformed cancer cells (Figure 1 and refs. 43, 44). Preclinical studies on various pancreatic cancer mouse models demonstrated that KRAS G12D siRNA could be effectively transferred to the tumor site, induce prominent regression in tumor size, and prolong survival, holding remarkable potential for PDAC treatment (43, 45). In 2020, a phase I clinical trial of iExosomes (NCT03608631) started with PDAC patients at various stages.

Gene editing: what is the true potential?

Kim et al. used doxycycline-inducible CRISPR/Cas9 as a therapeutic tool to target KRAS G12V, G12D, and G13D in CRC cells both in vitro and in vivo, demonstrating that a 7.2-fold reduction in tumor volume was achieved by the knockdown of G12V mutant by highly specific KRAS G12V single-guide RNA (sgRNA) in a xenograft model without altering the wild-type allele (Figure 1 and ref. 46). Although the proof-of-concept CRISPR applications promise allele-selective cancer treatment potential, there is still a lack of sufficient clinical data regarding safety and feasibility. A phase I study of ex vivo CRISPR/Cas9–edited patient-derived T cells has been conducted with a limited number of cancer patients (47–49). The clinical outcome of CRISPR/Cas9 PD-1–edited T cells in NSCLC patients (NCT02793856) corroborated the findings of another clinical study (48), supporting this method as a means of safe and feasible treatment. Yet the low treatment efficacy should be resolved in future trials (47).

RNA-editing CRISPR/Cas systems are coming into play for targeted cancer therapy, too (50). A growing body of in vitro studies indicates that CRISPR/Cas13a provides a powerful tool for interference with and editing of oncogenic transcripts in cancer cells (51, 52). It is especially remarkable that significant and specific knockdown of the KRAS G12D allele, with no influence on the wild-type allele, in pancreatic cancer cells was accomplished and inhibited tumor growth in vitro and in xenograft mouse models (52). With the rapid development of gene-editing systems, it seems very likely that engineered RNA-editing CRISRP/Cas will take its place in cancer therapy in the near future.

## Cancer immunotherapy: vaccines

**Figure 2:**
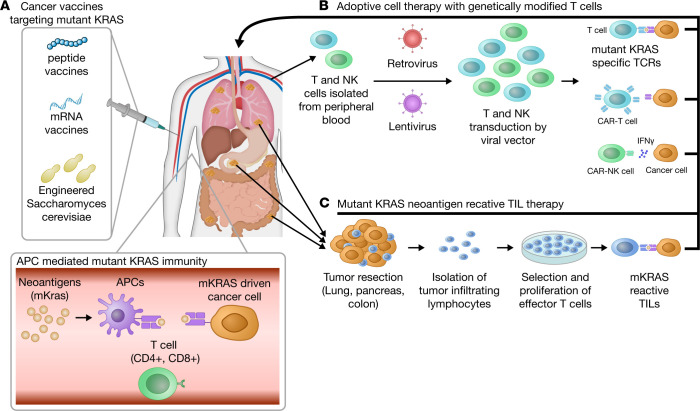
Immunotherapy regimens for the treatment of oncogenic KRAS–driven tumors. (**A**) Vaccines that promote oncogenic KRAS antitumor immunity. Peptide-, mRNA-, and DC-based vaccines can be administered to patients with lung, pancreatic, and colon cancer. Vaccines provide oncogenic KRAS neoantigens to MHC molecules and aim to develop cancer-specific long-term memory T cells. Upon tumor growth, activated T cells destroy cancerous cells through TCR-MHC binding. (**B**) Adoptive cell therapy with engineered T and NK cells. T and NK cells isolated from a patient’s blood are genetically modified by viral vectors to express specific T cell receptors (TCRs) and neoantigen specific NK receptors for a better recognition of oncogenic KRAS–expressing cancerous cells. Peripheral blood T cells from patients with lung, pancreatic, and colon cancer are alternatively used to create CAR-T and CAR-NK cells that express patient-specific, KRAS-driven cancer cell neoantigens. (**C**) After surgical resection of a tumor, patient-specific tumor-resident active T cells (TILs) are isolated and expanded and selected ex vivo. The most tumor-specific and functionally enriched T cells are administered to the patient intravenously after lymphodepletion.

Cancer vaccines provide tumor-specific treatment opportunities for KRAS-driven carcinogenicity. The generation of specific targeted immune responses may overcome the necessity to inhibit oncogenic KRAS via small-molecule inhibitors. There are multiple ways to develop cancer-specific immune responses, including multipeptide, dendritic cell–based, and mRNA-based vaccines (Figure 2 and ref. 6).

### Peptide-based vaccines.

Neoantigens are utilized to stimulate strong and tumor-specific immune responses by the peptide vaccines, which can be designed by variation of length, copy number, and peptide combinations (53). They can be identified from patients’ biopsies via sequencing and bioinformatic analysis for the delivery of most suitable personalized peptide-based vaccine (54). For example, Arbelaez et al. used synthetic long peptides (SLPs) harboring KRAS G12D mutatations alone or in combination, and examined their potential to elict TIL responses by using a lipoplex delivery system. While SLP with CpG adjuvants stimulated CD4^+^ T cells, the combination of lipoplexes and SLPs resulted in elevated stimulation of CD4^+^ and CD8^+^ T cells and better tumor suppression (55).

### DC-based vaccines.

The use of professional antigen-presenting cells (APCs), such as dendritic cells (DCs), is another vaccination method. DC-based vaccines have been used in the orthotopic PDAC murine model and resulted in an elevated CD8^+^ T cell response, specific lysis of tumors, and an increased number of IFN-γ–secreting T cells. Combined with a chemotherapeutic agent, DC vaccination caused complete elimination of the tumor (56). DCs were able to take up antigens produced by recombinant yeast and to undergo maturation. *Saccharomyces cerevisiae* was genetically engineered to express a unique combination of the seven most common RAS mutations observed in human cancers. This unique product, GI-4000, contains a heat-inactivated, engineered, intact yeast vaccine containing mutant KRAS peptides. In a phase II study of patients with NSCLC, GI-4000 was found to be immunogenic and tolerable (57). GI-4000 has been under further investigation in active clinical trials, including for pancreatic cancer (NCT03329248, NCT03387098, NCT03586869, NCT03136406), colorectal cancer (NCT03563157), squamous cell carcinoma (NCT03387111), and triple-negative breast cancer (NCT03387085).

### mRNA vaccines.

mRNA vaccines have the capacity to induce immune responses by expressing antigens without the need for genomic modification of cells and by allowing multiple antigens to be expressed at once (6). mRNA vaccine technology utilizes the recipient’s biological processes for protein translation (58). Moreover, mRNAs also function as adjuvants through their activation of pattern recognition systems (59). In 2016, Moderna and Merck announced their collaboration on a combinatorial therapy that contains Moderna’s mRNA-based personalized cancer vaccine with Merck’s PD-1 inhibitor pembrolizumab. According to their press release, a single mRNA was engineered to encode all of the patient’s specific mutations to be given in combination with pembrolizumab, which is expected to launch the most robust immune response against cancer (60). Presently, mRNA-4157 combined with pembrolizumab is being evaluated in 150 patients with melanoma as part of a phase II study (NCT03897881). The reasoning behind the combination of the mRNA cancer vaccine with a chemotherapeutic drug is that the duration of mRNA in the body necessary to create an adequate immune response is unknown. Furthermore, the combination of different therapies is expected to remove the barriers created by cancer cells to activate the immune system. In 2018, the two companies expanded their collaboration and made a novel shared-antigen vaccine therapy called mRNA-5671, which contains the four most prevalent KRAS mutations (G12D, G12V, G13D, and G12C) in solid tumors. In preclinical trials, mRNA-5671 elevated CD8^+^ T cell responses to mRNA encoding mutant KRAS. A phase I clinical trial of mRNA-5671 is currently being conducted in two arms, given as monotherapy or in combination with pembrolizumab (NCT03948763), in a total of 100 patients with lung, pancreatic, and colorectal cancer (61).

## Cancer immunotherapy: immune checkpoint inhibitors

Oncogenic KRAS signaling suppresses antitumor immunity by inducing TME reprogramming (62, 63), recruiting immunosuppressive cells, inhibiting T cell function (64), upregulating immune checkpoint molecules on tumor-infiltrating lymphocytes (TILs) and cancer cells, altering cytokine secretion, degrading enzyme and growth factor production (65), and downregulating MHC-I expression on APCs (66). Overall, oncogenic KRAS signaling initiates molecular events that lead to cancer cell immune evasion (67). Hence, most cancer patients with KRAS mutations exhibit resistance to various cancer therapies, leading to poor clinical outcomes. Monoclonal antibody (mAb) immunotherapies that block suppressive immune checkpoints have long been in use to enhance cancer immune surveillance and anticancer T cell activity. There are eight FDA-approved mAb products targeting immune checkpoints, such as CTLA-4, PD-1, PD-L1, and PD-L2, that dominate the immunotherapeutic market to treat some solid cancers and hematological malignancies (68). These modalities exhibit only moderate success rates in solid-cancer patients with KRAS mutations, even when combined with standard therapies. Indeed, only certain subgroups of patients with metastatic NSCLC, PDAC, and CRC are treated with the limited number of these FDA-permitted mAbs, since some genotypic and phenotypic features of specific subgroups are known to facilitate antitumor immune responses (69–72). Thus, much of the preclinical research in this area has focused on identifying novel co-immunomodulatory targets, while preclinical and clinical trials for inhibitory CD47, TIM-3, LAG-3, TIGIT, VISTA, and costimulatory 4-1BB have been ongoing for solid tumors, including the ones with KRAS mutations (65). High expression levels of inhibitory coreceptors were found to correlate with poor clinical outcomes, and coinhibition of these receptors has been shown to enhance cytotoxic T cell activity, T cell proliferation, and cytokine production (65, 73–76). Recent data from combinatorial treatment with AMG510 and anti–PD-1 immunotherapy increased survival of mice, and 90% of mice responded to therapy with complete tumor regression, suggesting that the efficiency of classical immunotherapies can be augmented as a result of the unleashing of antitumor immunity when oncogenic KRAS or downstream signaling pathways are inhibited (22). Furthermore, clinical findings in patients with lung cancer given AMG510 and anti–PD-1/PD-L1 in combination (NCT03600883) indicate that KRAS G12C inhibition sensitizes the TME and enhances TIL infiltration into the tumor site, increasing the efficacy of immunotherapy.

Immunotherapeutic and cancer vaccine combinations represent a viable alternative to monotherapies. As such, pembrolizumab has been combined with a GVAX (GM-CSF gene vaccine) regimen with cyclophosphamide (NCT02981524) for metastatic colorectal cancer patients with mismatch repair proficiency, and ipilimumab has been administered with or without GVAX for treatment of locally advanced, unresectable or metastatic PDAC (NCT00836407). Although pembrolizumab with GVAX was not clinically successful, partial biochemical responses from a subset of patients imply that GVAX still has a potential to modulate the TME if used in combination with other immunotherapies (77). Moreover, this approach improved the diversity of the peripheral T cell receptor (TCR) repertoire of patients and was associated with longer survival (78). Several studies have focused on immune modulation by oncogenic KRAS and checkpoint inhibitors in lung, pancreatic, and colon cancers, which are reviewed elsewhere (74, 79, 80).

## Adoptive cell therapy

Insufficient infiltration, dysfunction of TILs, and tumor evasion from T cell surveillance within the KRAS-driven tumor niche are well-known challenges in classical cancer therapy regimens (81–83). An alternative method for tackling these problems is to use patients’ TILs for T cell therapy (Figure 2), which has been a common practice in clinics for years. A pool of TILs isolated from a patient’s solid tumor are activated and expanded ex vivo and given back to the lymphodepleted patient, achieving clinically meaningful results without causing significant toxicity (84). A more elegant but slightly more complicated adoptive T cell therapy consists of sorting out T cell clones that explicitly target cancer neoepitopes with the highest persistence and reactivity from the patient’s TIL pool and expanding this population ex vivo before infusing it into the patient. This treatment method yielded significant tumor regression and clinical improvement in patients with KRAS G12D metastatic CRC and metastatic melanoma (85–87). Besides cancer neoantigen specificity, effective antitumor activity of adoptive TILs also relies on MHC serotypes on the tumor surface. As shown by Tran et al., the metastatic lesion in the lungs of a CRC patient evaded adoptive KRAS G12D–reactive TIL surveillance, demonstrating resistance to therapy. Deep sequencing revealed that the tumor had lost a copy of chromosome 6 encoding HLA-C*08:02 so that adoptive TILs could not recognize the tumor, indicating the importance of HLA-restriction element expression (85). Despite extensive applications, clinical outcomes imply that adoptively transferred unmodified TILs do not persist in vivo, hence failing to provide efficacious and durable treatment (88, 89).

Today, advanced molecular techniques make it possible to utilize highly specific and potent immune cells to recognize and kill cancer cells more efficiently. There are three different applications in use: (a) engineered TCR therapy, (b) chimeric antigen receptor T (CAR-T) cell therapy, and (c) NK cell therapy (90).

TCR therapy involves autologous patient TILs engineered to express TCRs that recognize specific tumor-associated antigen(s) (91). This technology allows T cells to target multiple neoepitopes simultaneously, facilitating the prevention of tumor evasion. One comprehensive study performed by Wang et al. identified specific TCRs highly reactive to KRAS G12D– and G12V–driven tumors in HLA-C*11:01 transgenic mice immunized with G12V and G12D KRAS peptides (92, 93). Murine PBMCs transduced with reactive TCRs that recognize HLA-C*11:01–expressing pancreatic cancer cells were evaluated in a pancreatic cancer xenograft mouse model. A significant reduction in tumor burden underlined the importance of TCR profile and the binding kinetics between HLA-C and TCR interaction in the specific, persistent, efficient TCR therapy.

Obstacles to adoptive TIL and TCR cell therapies, especially cancer cell evasion due to downregulation or loss of antigen and MHC expression, shifted adoptive cell therapy efforts to CAR-T cells (94). CAR-T cells were introduced to the field to bypass MHC/TCR–mediated T cell responses in cancer cells (95). It appeared as a valuable opportunity to treat clonal neoantigen–presenting tumors with engineered polyclonal neoantigen–reactive T cells without confronting any intratumoral heterogeneity (96–100). In the clinic, CAR-T cell therapy has been most successful in hematological malignancies, specifically C19-directed CAR-T cells (101). Substantial efforts have been made to implement CAR-T cell therapy for solid tumors. An increasing number of studies has revealed several neoantigens that may be used for CAR-T therapies for KRAS-driven cancers, including mesothelin (MSLN), carcinoembryonic antigen (CEA), HER2, MUC1, prostate stem cell antigen (PSCA), NK, and CD24 (95).

MSLN is a cell surface molecule upregulated in more than 80% of epithelial cancers, including PDAC and lung adenocarcinoma, and is correlated with tumor invasion and poor prognosis (102). After the induction of MSLN-specific CD8^+^ T cell responses was validated in preclinical studies (103–107), a phase I clinical trial of autologous redirected CAR-T cells expressing chimeric anti-MSLN and CD3-ζ with 4-1BB costimulatory domains was conducted with 16 patients with metastatic pancreatic cancer, yet only 2 of 6 patients showed stable disease (108). The poor clinical outcome was due to insufficient infiltration of the CAR-T cells into the tumor and dysfunction of the CAR-T cells over time.

To enhance treatment efficacy, CAR-Tmeso cells were reinforced with an oncolytic adenovirus expressing IL-2 and TNF-α and tested in a xenograft model of human PDAC in immunodeficient mice. The reinforced CAR-T cells exhibited improved sustainability, prolonged and enhanced antitumor T cell function, and higher efficacy in the primary tumor. Nevertheless, lung tumor metastasis could not be prevented (109). A phase I clinical trial using this approach has been recruiting patients since 2017 (NCT03323944). Human MSLN CAR-T cells (huCAR-Tmeso cells) have been applied to three cohorts stratified by lymphodepletion pretreatment and huCAR-Tmeso cell dose. Similarly, a phase I study (NCT03054298) of CAR-Tmeso cells for MSLN-expressing advanced solid cancers, including lung cancer, is still ongoing.

Carcinoembryonic antigen (CEA) is also a valuable target for adoptive cell therapy, as this glycoprotein family is expressed in nearly 75% of pancreatic cancers, and its upregulation is correlated with KRAS-driven metastatic CRC (110–112). As CEA is expressed on healthy epithelia of the gastrointestinal tract and the lung, anti-CEA CAR-T cell therapy in patients with metastatic CRC caused severe autoimmune responses (113). Chmielewski et al. showed that second-generation anti-CEA CAR-T cells that expressed CD3 and CD28 costimulatory domains persisted long-term in the tumor site, leading to a 67% tumor regression in an orthotopic mouse model of pancreatic cancer (114). Albeit promising in preclinical studies, CEA targeting did not provide the expected success in the phase I dose-escalation trial. Even under the influence of IL-2, seven patients displayed stable disease, and the other six patients exhibited disease progression (CRUKD/07/064). Moreover, a second dose provoked pulmonary toxicity; thus the trials were terminated (115). Consequently, there is an urgent need to find the best and the most effective strategy to overcome challenges in CAR-T cell therapy. For instance, coadministration of checkpoint inhibitors and targeting of intrinsic cell resistance to PD-1 mechanisms via dominant-negative receptor are two strategies that have been applied to enhance the efficacy of CAR-T cell treatments (116, 117). In another study, CD4^+^ and CD8^+^ T cells retrovirally transduced with a PD-1–CD28 fusion showed T cell activation, proliferation, and IFN-γ production when stimulated with recombinant PD-L1 and anti-CD3 (118). Moreover, CAR-T cell therapy that targets stroma is under investigation. Heparinase-overexpressing CAR-T cells implemented on solid tumors were shown to successfully degrade extracellular matrix, ameliorating T cell infiltration with persistent antitumor activity (119). Lo et al. targeted fibroblast activation protein (FAP) to decrease pancreatic tumor vascular density and to reduce extracellular matrix protein deposition (120). The adoptive transfer of FAP–CAR-T cells restrained the growth of desmoplastic human lung cancer xenografts, syngeneic murine pancreatic cancers, and autochthonous pancreatic cancer growth in an immune-dependent fashion (120). While tumor stroma-targeting strategies are widely applied, the benefits of stroma modulation is still controversial, as stroma degradation can favor aggressive dissemination, especially for pancreatic cancer (121, 122).

In recent years, there has been growing interest in using NK cell–based CAR therapy, due to advantageous biological features of NK cells, such as multiple mechanisms to activate cytotoxic activity, superior safety, minimal cytokine release syndrome, and easy generation from multiple sources (123, 124). NK cell–based therapeutic strategies include the receptor-mediated activation of NK cells, CAR engineering (CAR-NK), adoptive immunotherapy using donor-transformed NK cells, and augmentation of antibody-dependent cellular cytotoxicity (125). Using the KPC mouse model of PDAC, Hu et al. demonstrated that adoptive NK cell therapy elicits delayed tumor growth due to the release of highly potent IFN-γ compared with control NK cells (126).

Future perspective: what obstacles remain for targeting oncogenic KRAS?

In recent years, major steps have been taken in the battle against mutant KRAS–driven cancers. In particular, new technologies in the design of molecular therapies as alternatives to small-molecule drugs and classical therapies that indiscriminately kill cancer and healthy cells (Table 1) have shown promise. These new therapy regimens enable KRAS-driven cancers to be fought in various ways. Early in development, the novelty of RNAi was restricted by chemical and physiological limitations, including stability, in vivo delivery formulations, and biological barriers, such as enzymatic degradation by serum endonucleases and RNases, rapid renal clearance, membrane impermeability, and immune system activation (127, 128). Recent advances in RNA structure chemistry have made this tool more powerful in terms of improved stability and specificity. In addition, nonviral molecular systems have been introduced that use nanoscale liposome- or polymer-based formulations to provide specific, safe, and efficient delivery of RNAi molecules to tumors.

Progress in adoptive cell therapy enables immune cells to be armed with robust multi-weapons that recognize and kill cancer cells expressing mutant KRAS–specific neoantigens, thereby overcoming immune evasion, low efficacy, and specificity hurdles. CAR technology has been rapidly improving since it was first established; fourth-generation CAR-T cells express not only the single-chain variable fragment and immunoreceptor tyrosine–based activation motif but also costimulatory molecules for robust T cell activation. Nevertheless, T cell exhaustion, suppressive influence of the TME, unwanted immune responses to adopted T cells, and challenges in making large-scale, high-quality, clinical-grade products are the main issues that need to be resolved for this approach to be commonly applied. Notably, the use of CAR-NK cells seems to be an alternative compensating therapy, which attacks tumors in an MHC- and tumor antigen–independent manner while creating fewer complications. Although some progress has been made, further research is needed for broad application of CAR-NK therapy in the clinic. This approach might become a first line of treatment for KRAS-mutant solid tumors, because it is safer for clinical use and not restricted to the patient’s own blood as a resource. Monoclonal antibodies targeting immune checkpoints have opened up a new era in cancer treatment, but the low to modest efficacies of these regimens, especially in pancreatic and colon cancers, have failed to meet expectations. Nevertheless, recent preclinical and clinical data suggest that immune checkpoint blockade exhibits synergistic efficacy when combined with either adoptive cell therapies or inhibition of immunosuppressive molecules like FAK (22) in the TME. Consequently, the next step to elicit clinically meaningful responses in oncogenic KRAS–driven cancers is to find the safest and the most efficient combinations of drug and therapy regimens based on the genetic background of the patient. Also, intratumoral and interpatient heterogeneity in KRAS-driven tumors should be considered.

With the development of the mRNA vaccine technology, the ability to detect specific mutations in patient tumors through next-generation sequencing has carried the cancer vaccine concept to an advanced stage. Regarding the augmented T cell response in phase I trials, mRNA vaccine studies were expanded to target shared antigens of KRAS as well as patient-specific mutations and moved to the clinical stage. Owing to the success of stable coating and delivery of mRNA vaccines developed for COVID-19, as well as proof of their ability to stimulate robust immune responses, such as increased T cells and antigen-specific antibody stimulation, mRNA vaccines have attracted a great deal of interest in the industry for cancer treatments and caused a dramatic rise in the market value of the technology. We must yet await the actual role that mRNA vaccines will play in the treatment and prevention of KRAS-driven cancers.

## Concluding remarks

More than 30 years of intensive efforts to target oncogenic KRAS signaling with small-molecule drugs in clinical and preclinical studies showed that more efficient, more specific, and less toxic treatment methods are still needed. In recent years, advances in molecular technologies and growing cumulative knowledge regarding oncogenic KRAS have given rise to new and more potent therapeutic approaches. RNAi and CRISPR/Cas9 have been shown to be clinically safe and able to inhibit mutant KRAS and its regulatory miRNAs in a specific manner, with fewer off-target effects. This specificity drastically diminishes most health concerns and holds significant therapeutic potential. In recent decades, the need to solve therapeutic RNAi delivery and stability problems has been met with the development of nanosized, lipid- and polymer-based formulations. These fine-tuned RNAi therapeutics with versatile delivery systems can induce cancer cell apoptosis, halt tumor growth, and reduce tumor size, as seen with siG12D-LODER in PDAC treatment. Reduced tumor size was achieved in preclinical studies, implying that this approach can be channeled to personalized treatment strategies. On the other hand, CRISPR/Cas9–based animal cancer models and in vivo genome-wide gene screens are gaining substantial attention. Nevertheless, the clinical application will not occur until sufficient preclinical data are obtained.

Vaccine-based approaches targeting neoantigens in cancer cells have been improved in several ways. While multipeptide vaccines with scrambled KRAS mutants provoke cellular immunity, DC vaccines facilitate the emergence of a high number of IFN-γ–secreting lymphocytes. mRNA vaccines are getting more and more attention as more data become available. Moderna’s mRNA-5671, which is almost at the end of phase II, provides an antitumor immune response and treatment opportunity by targeting Kras (G12/V/C/G13D) neoepitopes without the necessity of maturation, editing, exogenous peptide production, or expansion of cells. Nevertheless, the biggest challenge has yet to be solved, as tumor neoantigens exhibit significant heterogeneity among individual patients with different immune responses. This challenge may be overcome by immune checkpoint inhibitors to halt immune suppression. Although some promising clinical results are available for monotherapies, these have not yielded much clinical success in more intricate and malignant cancer types, such as pancreatic cancer, because of the tumor’s low antigenicity and the immunosuppressive TME. For this reason, TME reprogramming by oncogenic KRAS signaling should be studied extensively for preventing T cell dysfunction. The driving factors for CAR-T cell exhaustion need to be studied in detail to facilitate antitumor immunity. Ultimately, emerging CAR-T variants that simultaneously target both the microenvironment and the tumor are likely to be used for personalized therapies in the near future. In conclusion, new cancer therapy alternatives against oncogenic KRAS provide safer, less toxic, less off-target, and more patient-specific treatments than classical therapies and several small-molecule drugs. The acquired resistance of KRAS-driven tumors to immunotherapy and drugs seems to be overcome by such multilevel personalized therapies when they are combined with the right classical treatments.

## Author contributions

HA and IED wrote, edited, and prepared original draft. UE, NCC, ME, AD, and MG wrote and contributed to figure drawings. EAS reviewed the manuscript. RI, HF, GOC, and IED supervised the preparation of the manuscript.

## Figures and Tables

**Table 1:**
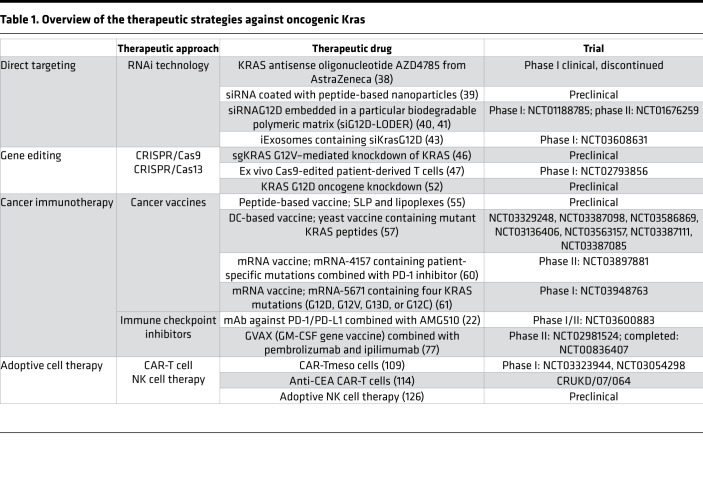
Overview of the therapeutic strategies against oncogenic Kras

## Version 1 01/11/2022

Electronic publication
